# Sequestration of plant defenses by spotted lanternfly (*Lycorma delicatula*) and effects on avian predators

**DOI:** 10.1007/s10886-025-01647-6

**Published:** 2025-10-23

**Authors:** Anne E. Johnson, Allison Cornell, Fang Zhu, Ashley E. Shay, Gabrielle Davis, Kelli Hoover

**Affiliations:** 1 Department of Entomology, The Pennsylvania State University, Philadelphia, PA 16802 USA; 2 Department of Biology, Penn State Altoona, Altoona, PA 16601 USA; 3 Huck Institutes of the Life Sciences, The Pennsylvania State University, University Park, Philadelphia, PA 16802 USA; 4 Ronald E. McNair Scholars Program, J. Jeffery and Ann Marie Fox Graduate School, The Pennsylvania State University, University Park, Philadelphia, PA 16802 USA

**Keywords:** Invasive species, Behavior, Birds, Quassinoids, Aposematic coloration

## Abstract

*Lycorma delicatula* is a polyphagous phloem feeder, which is spreading rapidly in the U.S., and is a major threat to grapes, hops, and ornamentals. Among its hosts, *L. delicatula* prefers *Ailanthus altissima*, an invasive tree from which it sequesters toxins, including several quassinoid compounds. We tested how these defenses affected predation by birds of nymphal and adult *L. delicatula*. Freeze-killed nymphs of each instar were reared with or without access to *A. altissima* and placed into cups on top of nest boxes occupied by house wrens (*Troglodytes aedon*). House wrens ate or fed their chicks a greater proportion of *L. delicatula* nymphs that had not had access to *A. altissima* than the nymphs that had access to this host plant. Adult *L. delicatula* reared with or without access to *A. altissima* were ground up and incorporated into separate batches of suet that were placed into double sided feeders set up at different sites during the winter. Trail camera video was reviewed to record how many times birds pecked each suet cake. Birds pecked the suet containing *L. delicatula* reared without access to *A. altissima* significantly more frequently than the suet containing adults that had fed on *A. altissima*. Of the quassinoids sequestered by *L. delicatula* from phloem sap of *A. altissima*, ailanthone and four other quassinoids were identified and quantified in different tissues, with the highest concentrations in the salivary glands. Results suggest that sequestration of toxic chemicals from *A. altissima* provides some protection to *L. delicatula* from avian predators.

**Supplementary Information:**

The online version contains supplementary material available at 10.1007/s10886-025-01647-6.

## Introduction

Spotted lanternfly (*Lycorma delicatula* (White) (Hemiptera: Fulgoridae) is an invasive planthopper that has dispersed widely from Berks County, Pennsylvania where it was first discovered, and is now established in at least 18 states as it spreads in all directions in the U.S. (NYSIPM 2024). Among its many hosts, *L. delicatula* shows a strong preference for tree of heaven (*Ailanthus altissima* (Mill.) Swingle (Sapindales: Simaroubaceae), with which it coevolved in Southeast Asia. *Ailanthus altissima* is an invasive plant in North America and in much of the world (Miller 1990; Barringer and Ciafré 2020). *Lycorma delicatula* obtains toxins from *A. altissima* through sequestration, a process in which animals acquire and store potentially harmful chemicals for their own benefit (Duffey 1980; Song et al. 2018; Beran and Petschenka 2022). *Ailanthus altissima* serves as a significant source of potential toxins for *L. delicatula*, producing both quassinoids and indole alkaloids, which have also been detected in this species (Polonsky and Fourrey 1964; Anderson et al. 1983; Souleles and Waigh 1984; Tang and Eisenbrand 1992; Xue and Yuan 1996; Bucar et al. 2007; Kim et al. 2011; Song et al. 2018). This planthopper likely uses these compounds to protect itself from avian predators and advertises its toxicity via aposematic coloration in the fourth instar and adult stages. One study found that wild magpies (*Pica pica* (L.) (Passeriformes: Corvidae) vomited after consuming field collected *L. delicatula* (Kang et al. 2011), while another showed that Oriental tits (*Parus minor* (Temminck and Schlegel) (Passeriformes: Paridae) ate more from butter balls containing *L. delicatula* that were collected from Korean willow (*Salix koreensis* Andersson (Malpighiales: Salicaceae) relative to those collected from *A. altissima*; this behavior was consistent with the concentration of the quassinoid ailanthone in adult *L. delicatula* (Song et al. 2018). In contrast to birds, a prior study found that arthropod predators were not deterred by sequestered defenses in any life stage of *L. delicatula* (Johnson et al. 2025).

Herein, we focused on the potential ability of all life stages of *L. delicatula* to sequester several quassinoids, including ailanthone, and how this affects predation by birds. Quassinoids are degraded triterpenoids and in nature are produced by plants in the family Simaroubaceae, which includes *A. altissima* (Curcino Vieira and Braz-Filho 2006). Quassinoids are cytotoxic and have been investigated for use in antimalarial, antiviral, anticancer, antiparasitic, antifungal, insecticidal, amoebicidal, and herbicidal purposes (Curcino Vieira and Braz-Filho 2006; Chen et al. 2021). These compounds taste bitter to humans and effectively deter herbivores from feeding on the plants that produce them (Curcino Vieira and Braz-Filho 2006), which suggests that quassinoids could similarly deter some types of predators from consuming herbivores that sequester them. One potential example of this is the Ailanthus webworm moth (*Atteva aurea* (Fitch) (Lepidoptera: Attevidae)), which was originally found in tropical and subtropical North and Central America where it feeds on native Simaroubaceous plants; it was able to extend its range as far north as Canada after the introduction of *A. altissima* by feeding on this host as well (Frank 2015). While evidence is limited, previous studies suggest that Ailanthus webworm is unpalatable to birds perhaps due to toxin sequestration (Evans 1983; Frank 2015).

In this study, we had several aims. First, we tested whether birds display a feeding preference for nymphal and adult *L. delicatula* that did not have access to *A. altissima* over those that did. Such preference would support the hypothesis that insects feeding on *A. altissima* are less palatable to birds, indicating that *L. delicatula* can sequester toxins from its preferred host plants, providing protection against bird predators. Additionally, we quantified the concentration of quassinoids in *L. delicatula* across life stages and host plants, which may influence its interactions with avian predators throughout development. Finally, we identified the insect tissues with the highest concentrations of quassinoids, providing insight into how these compounds may be stored and utilized for defense against different predators.

## Materials and methods

### Rearing of *L. delicatula*

In 2021, *L. delicatula* with or without access to *A. altissima* were obtained by rearing them several different ways over 2.5 years. Insects without access to *A. altissima* were reared in five 3 m x 2.4 m x 2.4 m outdoor enclosures made of PAK25 Anti-Insect Mesh (Hummert International, MO) over a frame made of 38 mm diameter galvanized steel tubes (described fully in Hoover et al. 2023) with access to planted silver maple (*Acer saccharinum* L. (Sapindales: Sapindaceae), weeping willow (*Salix babylonica* L. (Malpighiales: Salicaceae), black walnut (*Juglans nigra* L. (Fagales: Juglandaceae), and river birch (*Betula nigra* L. (Fagales: Betulaceae). In 2022 and 2023, *L. delicatula* without access to *A. altissima* were reared in 60.96 cm x 60.96 cm x 91.44 cm popup cages (Restcloud, Guangzhou China) and provided with potted grape (*Vitis vinifera* L. (Vitales: Vitaceae), strawberry (*Fragaria* × *ananassa* Duchesne (Rosales: Rosaceae), and cucumber (*Cucumis sativus* L. (Cucurbitales: Cucurbitaceae).

In 2021, *L. delicatula* that had access to *A. altissima* were obtained by rearing them in large outdoor enclosures, similar to those described above except that river birch was replaced with *A. altissima* (Hoover et al. 2023). In 2022, *L. delicatula* were also reared in popup cages like those described above except a potted *A. altissima* was added to the cages in addition to the other host plants. In 2023, *L. delicatula* with access to *A. altissima* were collected from *A. altissima* in Berks Co., PA as they progressed through their lifecycle; all insects were then frozen and stored at −80˚C until use. No insects were collected in the field to use for the no-Ailanthus treatments because we could not be certain they had not fed on *A. altissima* at some point in time.

### Quassinoid Chemical Analyses

To identify and quantify quassinoids present in *L. delicatula* individuals, we adopted a modified version of the Quick, Easy, Cheap, Effective, Rugged, and Safe (QuEChERS) method described in Lehotay (2006). Samples were frozen in liquid nitrogen and then homogenized with a Mr. Coffee Electric Coffee Grinder (Sunbeam Products, Inc., Boca Raton, FL). We then transferred 1 g of each sample to 15 mL centrifuge tubes and added 1 mL of 1% acetic acid in acetonitrile, 0.4 g of anhydrous MgSO4, 0.1 g of NaCl, 0.1 g of trisodium citrate, and 0.05 g of disodium hydrogen citrate. Samples were vortexed for 1 min and then centrifuged at 3,000 RCF for 10 min. One milliliter of supernatant from each sample was pipetted from the centrifuge tubes into dispersive solid phase extraction (dSPE) tubes, for which the selection process of which type of dSPE tube to use is described below. Samples were vortexed in the dSPE tubes for 1 min, then centrifuged again at 17,000 RCF for 10 min. Supernatant was pipetted into 1.5 mL microcentrifuge tubes and evaporated for approximately 1 h using a SpeedVac vacuum concentrator (ThermoFisher Scientific, Waltham, MA). Samples were resuspended in 100 µL of 1:1 acetonitrile: HPLC grade water and then sent to the Huck Institute’s Metabolomics Core Facility (RRID: SCR_023864) at Pennsylvania State University for LC-MS/MS analyses.

To ensure our QuEChERS process would result in clean samples with the least amount of quassinoid loss, we first tested four types of dSPE tubes by rearing *L. delicatula* adults without access to *A. altissima* in outdoor enclosures as described above and collecting sufficient insects for eight 1 g samples. We spiked each of these samples with 100 µL of a 1 µM solution of the following standards: ailanthone (≥98% pure), glaucarubinone (approximately 80% pure), 2’-acetylglaucarubinone (approximately 80% pure), 13,18-dehydroglaucarubinone(approximately 90% pure), and grandilactone A (approximately 70% pure) in acetonitrile. Of these, all but ailanthone (purchased from Sigma Aldrich, St. Louis, MO) were isolated from *A. altissima* and provided by Dr. Jakob Franke at University of Hannover, Germany. Each of these samples was run through the QuEChERS process with each type of dSPE tube being used to clean two samples (Catalog # 26125, 26219, 26216, and 26243, Restek, Bellefonte, PA). The average concentration of quassinoids across the two samples was compared to determine which dSPE tube resulted in the greatest return of quassinoids, which was catalog # 26219 containing 150 mg of MgSO_4_, 50 mg primary and secondary amine (PSA), 50 mg C18-EC (end capped), and 50 mg graphitized carbon black (GCB) for all five quassinoids. Thus, these dSPE tubes were used for processing all samples going forward.

To verify that other host plants did not contain quassinoids, samples were collected from silver maple, weeping willow, black walnut, river birch, and grape, with five 1 g samples of branch and leaf tissue that included phloem collected from each species and each sample was from a different plant. Phloem was collected from *A. altissima* grown in pots and in the field by snipping the stylets of feeding adult *L. delicatula* with dissection scissors and using capillary tubes to collect the droplets of phloem as they flowed out of the cut stylet, with five 1 mL samples collected from each plant (Fisher and Frame 1984). *Lycorma delicatula* with and without access to *A. altissima* were reared in popup cages in the greenhouse as described above or were field collected from *A. altissima*; five 1 g samples were collected of each life stage from each rearing plant species, including eggs of *L. delicatula* that had been reared in the same manner. *Lycorma delicatula* were not allowed to feed for 24 h after collection to allow time for phloem to be digested and waste excreted.

To determine the concentrations of quassinoids in different tissues, adult *L. delicatula* were field collected from *A. altissima* and dissected with tools cleaned with 70% ethanol under a microscope in 1x PBS in milliQ water. Tissues were divided into samples consisting of the cuticle including the wings, fat body, ovaries, gut, salivary glands, and remaining tissues (other), with the tissues from 10 *L. delicatula* pooled into each of five samples. Collected samples were cleaned using the modified QuEChERS method described above and then sent to the Huck Institute’s Metabolomics Core Facility (RRID: SCR_023864) at Pennsylvania State University for LC-MS/MS analyses.

For all LC-MS/MS analysis, samples (5 µL) were separated by a Thermo Vanquish Horizon ultra-high performance liquid chromatography (UHPLC) system with a Waters Acquity UPLC BEH C18 column (100 mm x 2.1 mm 1.7 µm particle size) maintained at 55˚C using a 20-min aqueous acetonitrile gradient at a flow rate of 250 µL/min. Solvent A was LC-MS grade water with 0.1% formic acid and Solvent B was LC-MS grade acetonitrile with 0.1% formic acid. The initial conditions were 3% B, increasing to 45% B at 10 min, and 75% B at 12 min where it was held at 75% B until 17.5 min before returning to the initial conditions. The eluate was delivered into the Thermo TSQ Quantis Plus mass spectrometer in single reaction monitoring mode (SRM) where the quassinoid standards ailanthone, glaucarubinone, 2’-acetylglaucarubinone, and 13,18-dehydroglaucarubinone were detected using heated electrospray ionization (HESI) in negative ion mode with a spray voltage of 2.9 kV. Grandilactone A, and the quassinoid precursor compounds quassin and neoquassin, were detected using HESI in positive ion mode with a spray voltage of 3.7 kV. Nitrogen gas flow rates were fixed with the sheath gas set to 50, aux gas to 10, and sweep gas to 1. Argon was used as the collision-induced dissociation gas (1.5mTorr). The ion transfer tube temperature was 325 °C and the vaporizer temperature was 350 °C. Optimal collision energies, product ions, and RF lens values were optimized for each compound by direct infusion (Table S1). Peak areas were calculated using Thermo Scientific Freestyle (version 1.8.63.0), which was then used to calculate the concentrations of each quassinoid in each sample. We generated standard curves correlating the peak areas to the concentration of standards of these compounds at 0 µM, 0.0016 µM, 0.008 µM, 0.04 µM, 0.2 µM, 1 µM, 5 µM, and 25 µM. Quassinoid standards were from the same sources as described for the dSPE tube testing, with the addition of quassin (≥98% pure) and neoquassin (≥98.0% pure) purchased from Sigma Aldrich (St. Louis, MO).

The total mean concentrations of quassinoids and for ailanthone alone as the predominant quassinoid were compared between phloem samples collected from *A. altissima* in the field, grown in pots in the greenhouse, and adult *L. delicatula* that were reared without access to *A. altissima*, in the greenhouse with access to *A. altissima*, or field collected from *A. altissima* using one-way ANOVAs. Two-way ANOVAs were used to compare the total mean concentrations of quassinoids and ailanthone alone in *L. delicatula* using insect source and life stage as independent variables. One-way ANOVAs were used to compare the total mean concentrations of quassinoids and for ailanthone alone among the tissues dissected from field collected adult female *L. delicatula*. Tukey HSD post hoc analyses were performed following ANOVAs that were significant at *p* < 0.05.

### Bird Predation Preference Assay Based on *L. delicatula* Diet

To test preferences of birds for *L. delicatula* with or without access to *A. altissima*, we set up 100 nest boxes around the Pennsylvania State University campus in University Park, PA, and those occupied by the most abundant bird species, house wrens (*Troglodytes aedon* Vieillot (Passeriformes: Troglodytidae)), were used in this study. Each nest box had a small cup attached to the lid to which we added 10 frozen *L. delicatula* nymphs that had access to *A. altissima* or not, and 10 live mealworms to draw the birds’ attention to the prey in the cups. Each nymphal life stage of *L. delicatula* was tested separately; adults were not tested because the fledglings left the nests several months before adult prey were available. Nest boxes were observed either by reviewing trail camera footage or, as we only had 6 trail cameras, with binoculars for an hour or until all nymphs were used to determine how the house wrens interacted with the provided insects and to be certain that prey items were not just being dropped on the ground. The cups were then checked after 24 h to count how many insects had been eaten; the proportions of consumed *L. delicatula* from each treatment were compared using a Chi-square test which, like all other statistical tests described in this paper, were performed in R (v. 4.3.1).

To test the preferences of birds based on adult *L. delicatula* diet, we made two batches of suet with *L. delicatula* reared in the outdoor enclosures described above, with one batch containing *L. delicatula* that had access to *A. altissima* and the other batch with those that had no access. The suet was made with a recipe from the Audubon Society with 30 g of ground *L. delicatula* added from one of the two rearing groups instead of bird seed (Audubon 2016). Suet was then placed into 6 double-sided suet feeders at three sites (2 per site) in central Pennsylvania. Each feeder had a suet cake from each treatment, which will henceforth be referred to as non-Ailanthus suet for the suet made with *L. delicatula* that did not have access to *A. altissima* and Ailanthus suet for the suet made with lanternflies that did; each suet feeder was monitored with a motion activated trail camera. The videos from these cameras were reviewed to record the number of pecks each bird made to each suet treatment when feeding, the species of the bird, and the start and end time of each visit.

Statistical analyses of suet feeder data were completed using random effects hurdle negative binomial models (α = 0.05) with the R package *glmmTMB* (Brooks et al. 2017). For the full dataset, we compared the number of times birds pecked the suet, with *L. delicatula* access to *A. altissima* during rearing considered a fixed effect and bird species visiting and the site of the feeder considered random effects, while the hurdle was used to remove excessive zeros as we did not know if zeroes were caused by genuine disinterest in the suet or another factor, such as the bird being scared away. As woodpeckers (Family Picidae) frequently pecked many times more than other birds, data were also divided into two groups, one containing woodpecker species and the other containing the remaining bird species. These data were then analyzed to compare the number of times the birds pecked the suet with the rearing treatment considered a fixed effect, bird species and site as random effects, and a hurdle to remove excessive zeros. For each of the three woodpecker species and the five most common non-woodpecker species, analyses to compare the number of times birds pecked the suet were performed individually, with the rearing treatment considered a fixed effect and site considered a random effect with a hurdle added to remove excessive zeros.

## Results

### Quassinoid Chemical Analyses

**Fig. 1:**
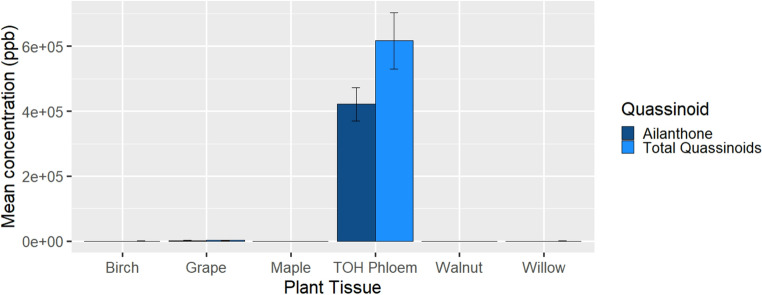
Average concentrations of ailanthone and total quassinoids in various host plants used for rearing *L. delicatula*

*Lycorma delicatula* and *A. altissima* phloem samples contained consistently higher concentrations of ailanthone than any other quassinoid (see Table S2 for full list of mean concentrations in different types of samples). Potted *A. altissima* on which *L. delicatula* were reared contained low concentrations of quassinoids relative to those found in phloem collected from *A. altissima* in the field in 2022, which contained a mean ailanthone concentration of 421,542 ± 50,979 ppb, while the average concentration of all other quassinoids combined was 195,034 ± 40,218 ppb in *A. altissima* phloem (Fig. 1).

**Fig. 2:**
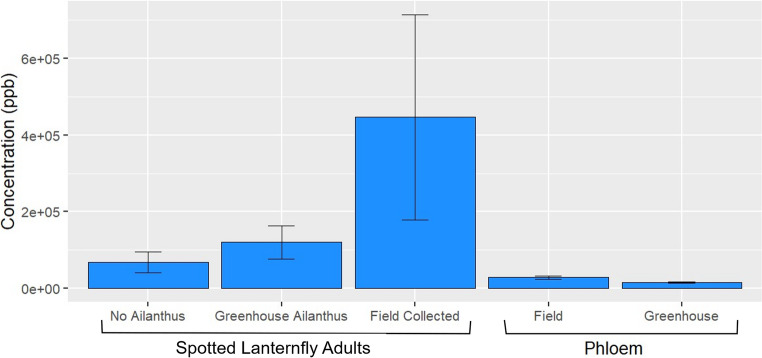
Mean total concentration of quassinoids found in adult *L. delicatula* that were field collected from *A. altissima*, reared in the greenhouse with or without access to *A. altissima*, and phloem collected from *A. altissima* in the field or grown in pots

Field collected adult *L. delicatula* had an average total concentration of quassinoids of 446,451 ± 267,653 ppb, adults reared with access to potted *A. altissima* had an average of 119,950 ± 42,890 ppb, and adults reared without access to *A. altissima* contained an average concentration of 68,286 ± 27,185 ppb. Phloem collected from *A. altissima* in the field contained an average total concentration of quassinoids of 28,413 ± 3,642 ppb and phloem from potted *A. altissima* contained an average of 15,646 ± 1,564 (Fig. 2). However, there were no significant differences in the mean total concentrations of quassinoids among these samples (F = 2.14, df = 4, 20, *p* = 0.113) when compared using a one-way ANOVA. There were also no significant differences in the mean total concentrations of ailanthone in these samples (F = 2.34, df = 4, 20, *p* = 0.0897) (Fig. S1, Table S2).

**Fig. 3:**
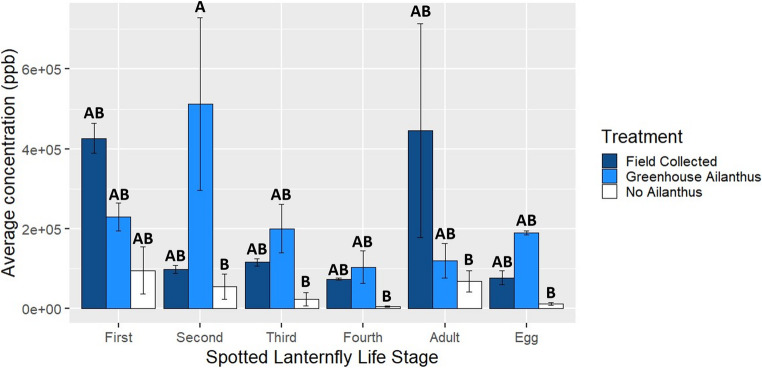
Mean total concentration of quassinoids found in *L. delicatula* field collected from *A. altissima* or reared in the greenhouse with or without access to *A. altissima* by life stage. Tukey HSD post hoc analyses were performed following ANOVA. There was no significant difference (*P* < 0.05) in the levels of concentration among the samples with the same alphabetic letter over the bar

There were statistically significant differences in the total concentrations of quassinoids based on how *L. delicatula* were reared (F = 8.12, df = 2, 72, *p* < 0.001), their life stage (F = 2.51, df = 5, 72, *p* = 0.0374), and the interaction between these variables (F = 2.46, df = 10, 72, *p* = 0.0138) when compared using a two-way ANOVA, unlike the comparison made with a one-way ANOVA between the concentrations in adult *L. delicatula* and *A. altissima* phloem (Fig. 3). *L. delicatula* reared without access to *A. altissima* had a significantly lower average quassinoid concentration than those reared in the greenhouse on *A. altissima* (*p* = 0.0013) and those that were field collected from *A. altissima* (*p* = 0.0046). Total average concentrations of quassinoids were significantly higher in second instar *L. delicatula* reared on *A. altissima* in the greenhouse than second (*p* = 0.0343), third (*p* = 0.0158), and fourth (*p* = 0.0098) instar nymphs reared without access to *A. altissima*, adults reared without access to *A. altissima* (*p* = 0.0470), and eggs of those reared without access to *A. altissima* (*p* = 0.0116) (Fig. 3). There were also significant differences in the average concentrations of ailanthone alone based on how *L. delicatula* were reared (F = 6.61, df = 2, 72, *p* = 0.0023) and the interaction between how they were reared and their life stage (F = 2.108, df = 10, 72, *p* = 0.0346). *L. delicatula* reared without access to *A. altissima* had a significantly lower average ailanthone concentration than those that were field collected from *A. altissima* (*p* = 0.0015). The average total concentration of ailanthone was significantly higher in field collected adults than first (*p* = 0.0316), second (*p* = 0.0276), third (*p* = 0.0115), and fourth (*p* = 0.0064) instar nymphs, as well as adults (*p* = 0.0275) and eggs (*p* = 0.0067) that were reared without access to *A. altissima* (Fig. S2). The average total concentration of ailanthone in field collected adults was also significantly higher than that in fourth instar nymphs (*p* = 0.0284), adults (*p* = 0.0250), and eggs (*p* = 0.0436) that were reared with access to *A. altissima* in the greenhouse (Fig. S2). The average total concentration of ailanthone in field collected adults was significantly higher than that in the field collected fourth instar nymphs (*p* = 0.0484) and eggs (*p* = 0.0328) (Fig. S2).

**Fig. 4:**
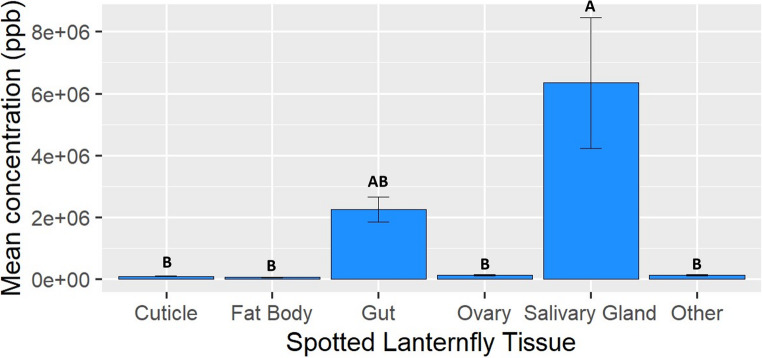
Mean total concentration of quassinoids found in tissues of adult female *L. delicatula* field collected from *A. altissima*. Tissue types with significantly different concentrations have different letters over the bar (*p* < 0.05)

There were significant differences in the average total concentrations of quassinoids among different tissues of *L. delicatula* adults (F = 4.22, df = 5, 24, *p* = 0.0068); salivary glands had a higher average concentration than samples containing cuticle (*p* = 0.0155), fat body (*p* = 0.0140), ovaries (*p* = 0.0160), and all other tissues combined except the gut (*p* = 0.0161) (Fig. 4). Average total quassinoid concentration was not significantly different in the gut than the category of all other tissues. There were also differences in average concentrations of ailanthone alone among different tissues (F = 113.1, df = 5, 24, *p* < 0.001); salivary glands had significantly higher average concentrations than those in the samples containing cuticle (*p* < 0.001), fat body (*p* < 0.001), gut (*p* < 0.001), ovaries (*p* < 0.001), and all other tissues (*p* < 0.001). Also, average concentrations in the gut were higher than those in the cuticle (*p* < 0.001), fat body (*p* < 0.001), ovaries (*p* < 0.001), and all other tissues combined (*p* < 0.001) (Fig. S3).

### Bird Predation Preferences Based on *L. delicatula*’s Access To A. Altissima

**Fig. 5:**
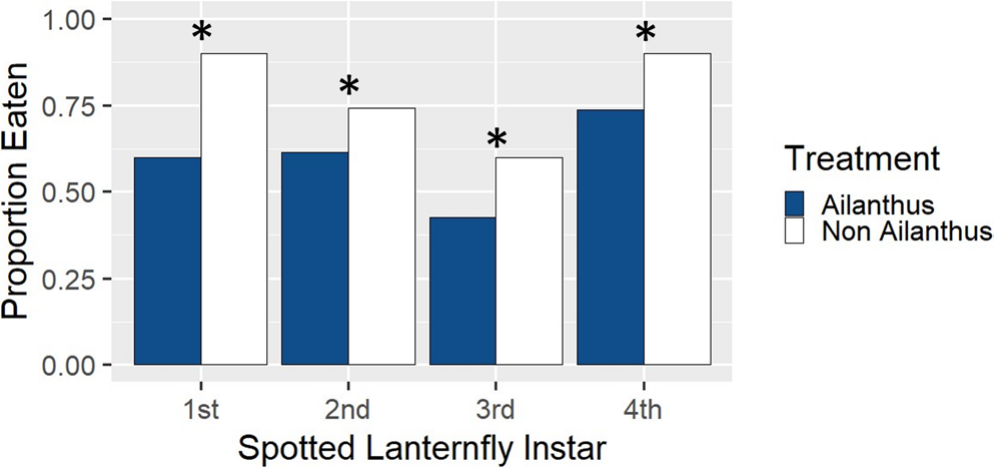
The proportion of *L. delicatula* nymphs reared with or without access to *A. altissima* by instar consumed by house wrens from feeding cups on top of nest boxes after 24 h. An asterisk over a set of bars indicates a significant difference between treatments within a life stage at *P* < 0.05

Nesting house wrens ate or fed to their chicks a significantly larger proportion of *L. delicatula* reared without access to *A. altissima* of every nymphal instar (Fig. 5). For first instars, a total of 16 nest boxes were occupied by house wrens that used 90% of the provided *L. delicatula* reared without access to *A. altissima*, which was significantly more than the 60% of first instars with access to *A. altissima* that were eaten (χ^2^ = 19.2, df = 1, *p* < 0.001). For second instars, a total of 28 nest boxes were occupied by house wrens that used 74.29% of the provided *L. delicatula* reared without access to *A. altissima*, which was significantly more than the 61.43% of second instars with access to *A. altissima* (χ^2^ = 5.31, df = 1, *p* = 0.02126). For third instars, a total of 30 nest boxes were occupied by house wrens that used 60% of the provided *L. delicatula* reared without access to *A. altissima*, which was significantly more than the 42.67% of third instars with access to *A. altissima* (χ^2^ = 9.02, df = 1, *p* = 0.0027). For fourth instars, a total of 14 nest boxes were occupied by house wrens that used 90% of the provided *L. delicatula* reared without access to *A. altissima*, which was significantly more than the 73.75% of fourth instars with access to *A. altissima* that were consumed (χ^2^ = 5.82, df = 1, *p* = 0.0159).

**Fig. 6:**
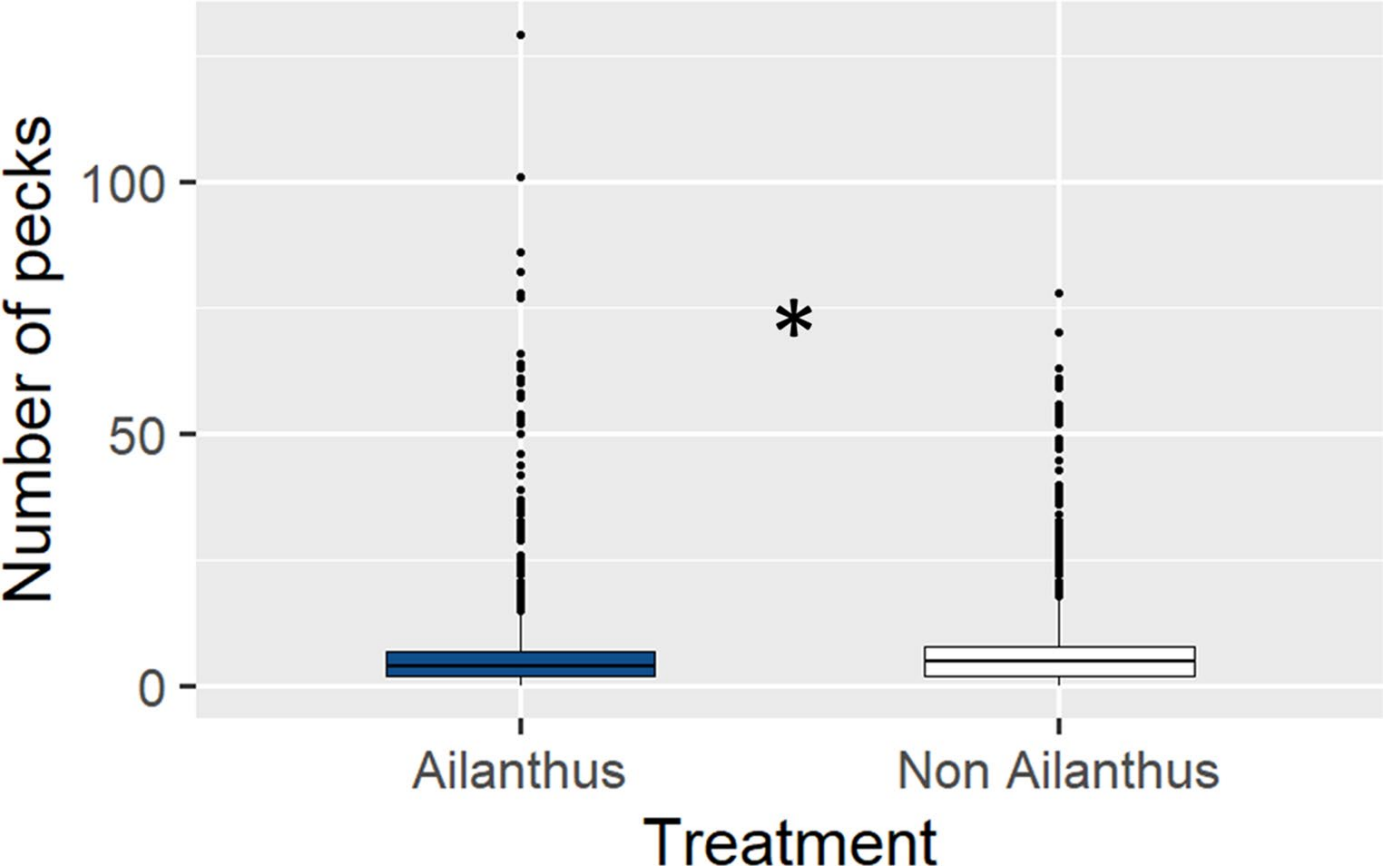
The number of times birds (woodpeckers and non-woodpeckers combined) pecked suet containing *L. delicatula* that did not have access to *A. altissima* relative to suet containing those that did. Suet containing *L. delicatula* reared without access to *A. altissima* was pecked significantly more times than those reared with access (indicated by asterisk, *p* < 0.05)

**Fig. 7:**
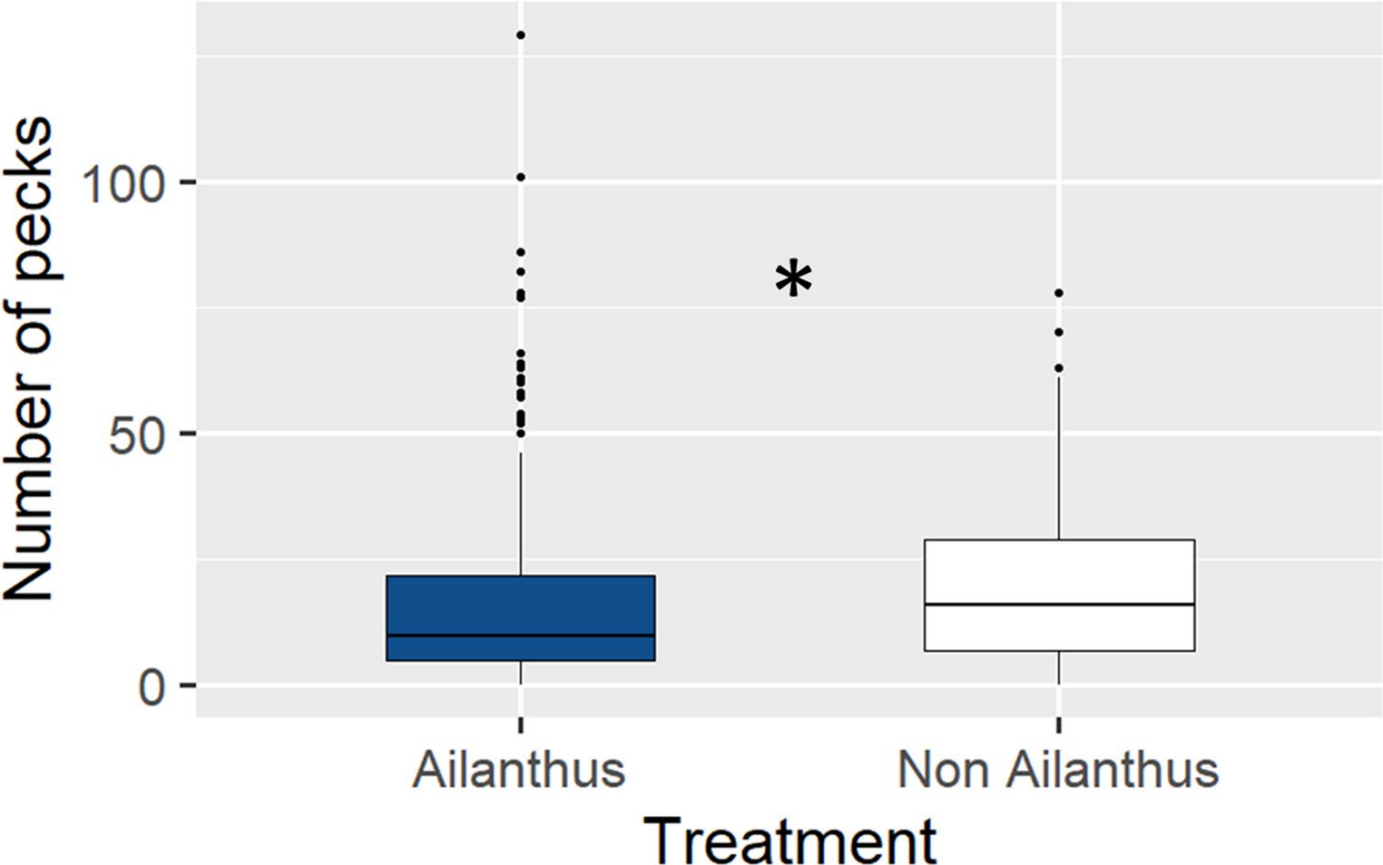
The number of times woodpeckers pecked suet containing *L. delicatula* that did not have access to *A. altissima* relative to suet containing those that did. Suet containing *L. delicatula* reared without access to *A. altissima* was pecked significantly more times than those reared with access (indicated by asterisk, *p* < 0.05)

**Fig. 8:**
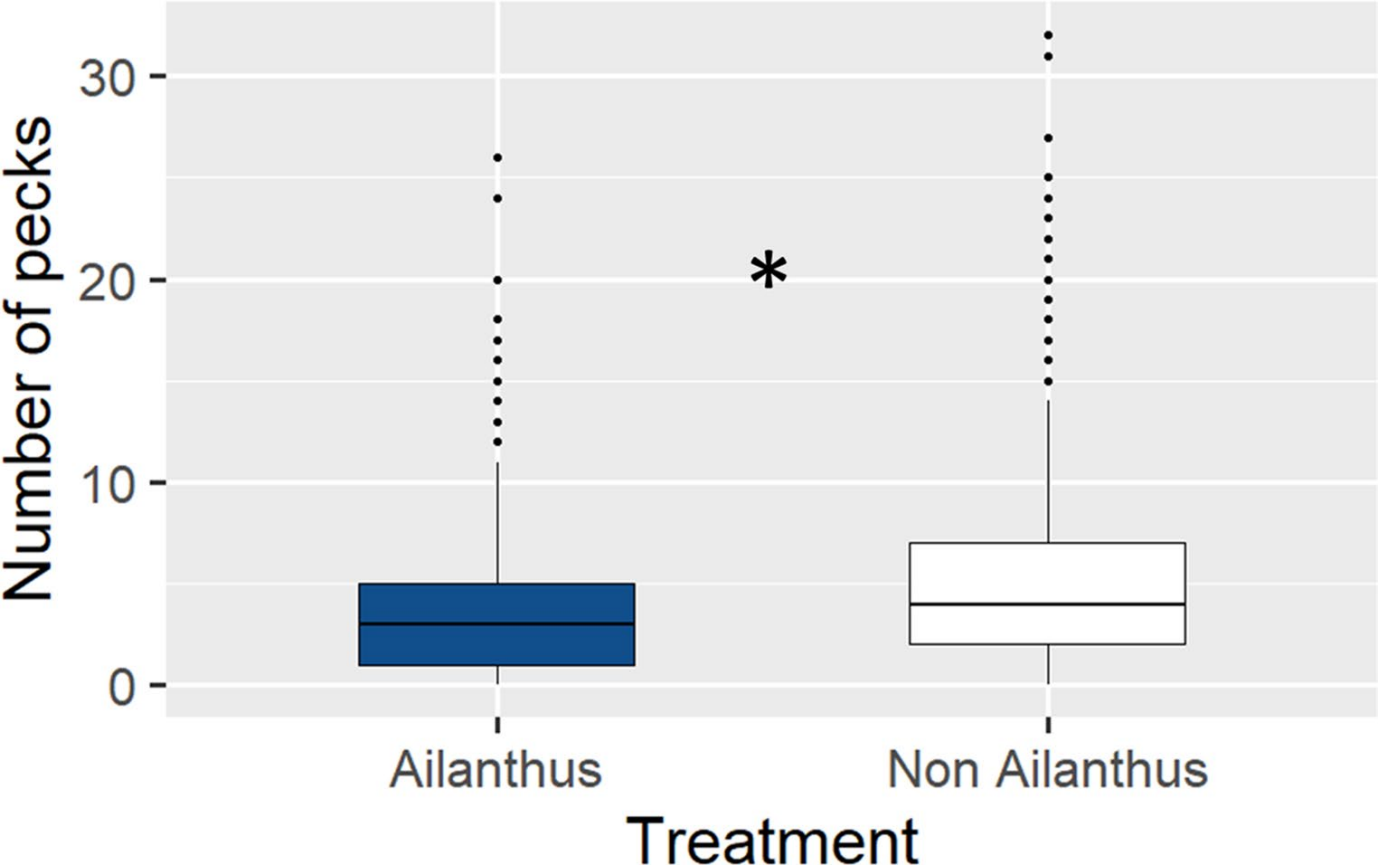
The number of times birds that were not woodpeckers pecked suet containing *L. delicatula* that did not have access to *A. altissima* relative to suet containing those that did. Suet containing *L. delicatula* reared without access to *A. altissima* was pecked significantly more times than those reared with access (indicated by asterisk, *p* < 0.05)

Birds also showed a preference for adult *L. delicatula* reared without access to *A. altissima* in our suet study. Across species, birds pecked the suet made with *L. delicatula* that did not have access to *A. altissima* significantly more than suet made with *L. delicatula* that had access (z = 8.163, *p* < 0.001); the non-Ailanthus suet was pecked an average of 6.87 times per visit vs. 6.2 times per visit when feeding on the Ailanthus suet (Fig. 6). Woodpeckers as a group also pecked the non-Ailanthus suet significantly more than the Ailanthus suet (z = 3.442, *p* < 0.001), (mean of 19.82 times per visit and 17.12 times per visit, respectively) (Fig. 7). In addition, birds that were not woodpeckers pecked the non-Ailanthus suet significantly more than the Ailanthus suet (z = 7.759, *p* < 0.001), (average of 5.11 times per visit and 3.86 times per visit, respectively (Fig. 8).

The three woodpecker species that visited the feeders, from the most common to the least common, were the downy woodpecker (*Picoides pubescens* (L.) (Piciformes: Picidae)), the red-bellied woodpecker (*Melanerpes carolinus* (L.) (Piciformes: Picidae)), and the hairy woodpecker (*Leuconotopicus villosus* (L.) (Piciformes: Picidae)). Both downy woodpeckers (z = 2.99, *p* = 0.0028) and red-bellied woodpeckers (z = 2.19, *p* = 0.0283) pecked the non-Ailanthus suet more than the Ailanthus suet, pecking an average of 20.72 times and 18.18 times, respectively (Fig. S4). Hairy woodpeckers did not show a significant preference between the types of suet (z = 1.646, *p* = 0.0997) (Fig. S4).

The five most common species of birds that were not woodpeckers, from most to least common, were black-capped chickadees (*Poecile atricapillus* (L.) (Passeriformes: Paridae)), tufted titmice (*Baeolophus bicolor* (L.) (Passeriformes: Paridae)), white-breasted nuthatches (*Sitta carolinensis* Latham (Passeriformes: Sittidae)), red-breasted nuthatches (*Sitta canadensis* L. (Passeriformes: Sittidae)), and Carolina wrens (*Thryothorus ludovicianus* (Latham) (Passeriformes: Troglodytidae)). Both black-capped chickadees (z = 6.82, *p* < 0.001) and white-breasted nuthatches (z = 4.07, *p* < 0.001) pecked the non-Ailanthus suet more than the Ailanthus suet, with pecking an average of 5.43 times and 3.94 times, respectively (Fig. S5).

## Discussion

To adapt to their toxic host plants, some insects sequester plant toxins in their bodies to defend against natural enemies (Beran and Petschenka 2022; Petschenka et al. 2022). In our study, we found that birds showed a preference for all nymphal stages and adults of *L. delicatula* that had not fed on *A. altissima*, suggesting the potential role of sequestration in defense of this species against predation by birds. While preference for adults not fed on *A. altissima* was expected and consistent with previous work (Song et al. 2018), preferences for nymphs that had not fed on *A. altissima* has not been previously reported. Our results contrast with the finding in Song et al. (2018) in which birds did not show a significant difference in the number of times they pecked butter balls that contained tissues from third instar nymphs that were field collected from Korean willow compared to *A. altissima*. One possible explanation for this is differences in tolerance for quassinoids between different bird species; Song et al. (2018) used Oriental tits while we tested house wrens. It is also possible that there is avian interspecific variation in tolerance or detection of quassinoids based on the differences we observed among different bird species in our suet choice experiment in which not all bird species showed a preference for suet made with *L. delicatula* without access to *A. altissima*. Another factor could be the difference in how *L. delicatula* were obtained; we reared *L. delicatula* in enclosures to be certain that they had never had access to *A. altissima*, while Song et al. (2018) used only field collected *L. delicatula*. Although the authors found no *A. altissima* near the Korean willows from which they were collected, nymphs frequently move between hosts and can travel tens of meters within a week (Liu 2019; Keller et al. 2020); *L. delicatula* are estimated to spread radially 38.6–46.2 km/year (Cook et al. 2021). Thus, it is possible that the nymphs reported in Song et al. (2018) could have fed on *A. altissima* at some point prior to collection, allowing them to sequester some toxins, reducing the difference in palatability between treatments. Additionally, the birds we used to test predation preferences for nymphal *L. delicatula* stages were raising nestlings during our tests, and therefore, could have been more selective in the prey they chose to utilize compared to other life stages. Several bird species prepare insect prey for their nestlings potentially to help their chicks avoid prey defenses, indicating that the life stage of birds can influence their feeding behaviors (Barba et al. 1996; Ponz et al. 1999). We observed that, of the nymphs that were used by house wrens, the parents sometimes ate both the *L. delicatula* reared with and without access to *A. altissima* but only fed their chicks nymphs reared without access to *A. altissima* (Davis and Johnson personal observations). This could indicate that parent birds were less willing to tolerate quassinoids in *L. delicatula* prey for their offspring than they were for themselves.

We found the quassinoids ailanthone, glaucarubinone, 2’-acetylglaucarubinone, 13,18-dehydroglaucarubinone, and grandilactone A in many of our *L. delicatula* and *A. altissima* samples. Among them, ailanthone is widely known to occur in *A. altissima* and the other quassinoids used for standards were isolated directly from *A. altissima*. However, quassin and neoquassin, which are precursors of the quassinoids we tested for, were not present in most of our samples. Ailanthone was consistently at the highest concentrations in the insects and *A. altissima* phloem samples (Table S2).

While not statistically different due to high variability within sample types, the average total quassinoid concentration appeared to be higher in adults than in *A. altissima* phloem, indicating that the insects acquired and sequestered quassinoids from phloem as they fed. The concentrations of quassinoids varied over the life cycle of *L. delicatula*, starting high in the earlier instars of all the rearing treatments and then declining throughout the nymphal stages before rising again and peaking in the adults reared with access to *A. altissima* and those that were field collected, while generally decreasing throughout the life cycle in those reared without access to *A. altissima* (Table S2; Fig. 2). These findings indicate that quassinoids found in *L. delicatula* come from sequestration from *A. altissima*, as we found no quassinoids in the alternative host plants we used for rearing, which was expected given that the other hosts are not in the Simaroubaceae. The increase in quassinoids in the adult stage also coincides with a shift in host plant preference to *A. altissima* documented in host choice studies (Murman et al. 2020). The higher levels of quassinoids in first instar nymphs, notably those reared without access to *A. altissima*, were likely passed to the eggs from their mothers, a capability observed in a variety of other insect orders including Lepidoptera, Coleoptera, and Orthoptera (Nishida 1994; Robert et al. 2017; Pugalenthi and Livingstone 1995). We also found quassinoids in both the ovaries and eggs of *L. delicatula*, which would be expected if quassinoids are transferred to egg masses during oviposition, providing the eggs with chemical defenses. This finding may contribute to the observed low frequency of predation of *L. delicatula* egg masses observed in the field (Johnson et al. 2025).

The concentrations of quassinoids reported here relate well to the observed predation behavior in birds feeding on *L. delicatula*. Notably, lower concentrations in the nymphal life stages are consistent with the previous study by Song et al. (2018) discussed above, where differences between birds’ reactions to nymphs based on diet were less pronounced than their reactions to adults. This is also consistent with our previous work in which community scientists reported that nymphal life stages were more likely to be eaten whole by predators than adults (Johnson et al. 2023). In addition, differences in the proportion of *L. delicatula* of different life stages used by our nesting house wrens showed a similar pattern to the differences in the quassinoid concentrations in *L. delicatula* based on whether they had access to *A. altissima* or not, supporting the hypothesis that the level of quassinoids in prey affects preferences of the birds feeding on them. Besides their high toxicity, the aversive taste of these quassinoids may also play a role in providing some protection of *L. delicatula* from avian predators (Duan et al. 2021; Petschenka et al. 2022). Over time, birds are capable of learning to avoid prey that are unpalatable, with visual, olfactory, and gustatory cues potentially allowing birds to learn to avoid *L. delicatula*. Notably, scent and taste could play a role in the protection of first through third instar nymphs, which lack the red coloring (aposematic coloration) of 4th instar nymphs and adults. We observed 332 mammalian visits to our suet feeders, of which 311 (2 cats, 203 mice, 5 raccoons, and 101 squirrels) were to the non-Ailanthus suet and lasted an average of 48 ± a standard deviation of 89.9 s each. In contrast, there were only 21 (14 mice and 7 squirrels) visits to the Ailanthus suet for an average of 14 ± 37.3 s each. Mammals that avoided the Ailanthus suet may have done so based on smell, which is noticeable to humans and is likely the case for other vertebrates as well.

The locations where quassinoids are stored within the bodies of *L. delicatula* could also affect the behavior of bird predators. In a recent community science project, it was observed that predators would often remove the wings of adult *L. delicatula* before feeding on them (Johnson et al. 2023). This could be a way for predators to reduce the amount of quassinoids that they are ingesting as there were quassinoids found in the cuticle, which is like behaviors seen in other predators that avoid feeding on the areas of their prey that contain toxins (Fink and Brower 1981; Glendinning 2007; Rafter et al. 2013). However, the wings may be discarded for other reasons, such as being less nutritious, as there are examples of birds removing the wings of moths and periodical cicadas which are not chemically defended (Steward et al. 1988; Barba et al. 1996; Ponz et al. 1999). As predatory birds in the US become more familiar with *L. delicatula*, they may develop other strategies to better tolerate chemically defended prey, such as by avoiding consuming tissues with high quassinoid concentrations.

Further work on sequestration by *L. delicatula* is warranted. First, we found that the concentrations of quassinoids were significantly higher in adult insects collected from the field than those reared in the greenhouse with or without access to *A. altissima*. This difference may be due to the higher concentrations of quassinoids in *A. altissima* phloem collected from the field relative to trees grown in the greenhouse. However, we did not find a significant difference when adults were compared to each other when comparing the concentration of quassinoids in phloem collected from *A. altissima* both in the field versus in the greenhouse, potentially due to small sample size and differences in power between the statistical tests. This difference is important to keep in mind in future experiments with *L. delicatula*, as it adds to the ways in which lab-reared *L. delicatula* have been noted to differ from those found in the field (Urban and Leach 2023), which may be another confounding factor to studies that use closed systems to rear *L. delicatula*.

It would also be worthwhile to investigate what our results mean for *L. delicatula* management, notably through conservation biological control (Shields et al. 2019). Arthropod predators show potential for use in augmentation or conservation biological control, as we have previously found that, unlike birds, arthropods do not show a preference for *L. delicatula* that have been reared without access to *A. altissima* (Johnson et al. 2025). Based on birds’ preference for *L. delicatula* that had never fed on *A. altissima*, the next step could be to explore whether removing *A. altissima* from an area enhances bird predation of *L. delicatula*, resulting in a reduction in its population. It would also be interesting to investigate how the age and health of *A. altissima* affects quassinoid production and, thus, the palatability of *L. delicatula* to predators. While birds in our experiments showed a preference for the *L. delicatula* that had been reared without access to *A. altissima*, they still fed on the nymphs and the suet made with adults that had access, which could have been influenced by the quality of the *A. altissima* provided to captive *L. delicatula*. If the *A. altissima* used for our captive-reared *L. delicatula* had lower quassinoid levels because the plants were younger and more stressed by being in pots, this could be good news for the application of conservation biological control tactics in the field, as prioritizing the removal of mature, healthy *A. altissima* may allow for higher predation rates.

## Supplementary Information

Below is the link to the electronic supplementary material.

ESM 1 (DOCX 2.40 MB)

## Data Availability

The data that support the findings of this study are openly available in ScholarSphere at https://doi.org/10.26207/qrrr-1d84.
