# Supplementary material for: Sequestration of plant defenses by spotted lanternfly (Lycorma delicatula) and effects on avian predators
Source: J Chem Ecol. 2025 Oct 23;51(6):102. doi: 10.1007/s10886-025-01647-6 (PMC12546476; doi:10.1007/s10886-025-01647-6)
Supplement: Supplementary file 1 — (DOCX 2.40 MB) [file 10886_2025_1647_MOESM1_ESM.docx]

**Supplementary Figures**

Johnson et al. (2025) Sequestration of quassinoids by spotted lanternfly (*Lycorma delicatula*) and effects on avian predators.

**Fig. S1** Mean concentration of ailanthone found in adult *L. delicatula* field collected from *A. altissima*, reared in the greenhouse with or without access to *A. altissima*, and phloem collected from *A. altissima* in the field or grown in pots. There were no significant differences in the average concentrations

**Fig. S2** Mean concentration of ailanthone found in *L. delicatula* field collected from *A. altissima* or reared in the greenhouse with or without access to *A. altissima* by life stage. Sample types with concentrations that were significantly different based on the interaction between the rearing treatment and life stage have different letters over the bar (p < 0.05)

**Fig. S3** Mean concentration of ailanthone alone found in tissues of adult female *L. delicatula* field collected off *A. altissima*. Tissue types with concentrations that were significantly different have different letters over the bar (p < 0.05). The y-axis uses a log_10_ scale

**Fig. S4** The number of times individual woodpecker species pecked suet containing *L. delicatula* that did not have access to *A. altissima* relative to suet containing those that did. Suet containing *L. delicatula* reared without access to *A. altissima* was pecked significantly more by downy woodpeckers and red-bellied woodpeckers than those reared with access (shown with an asterisk over the bars within bird species, p < 0.05)

**Fig. S5** The number of times the five most common non-woodpecker bird species pecked suet containing *L. delicatula* that did not have access to *A. altissima* relative to suet containing those that did. Suet containing *L. delicatula* that were reared without access to *A. altissima* was pecked significantly more by black-capped chickadees and white-breasted nuthatches than suet containing prey that were reared with access (shown with an asterisk over the bars, p < 0.05)

**Supplementary Tables**

**Table S1:** Details of relevant characteristics of LC-MS analysis of quassinoid compounds detected in *A. altissim*a and sequestered by *L. delicata.*

| **Compound** | **Retention Time (min)** | **RT Window (min)** | **Polarity** | **Precursor (m/z)** | **Product (m/z)** | **Collision energy (V)** | **RF lens (V)** | **Min dwell time (ms)** |
| --- | --- | --- | --- | --- | --- | --- | --- | --- |
| Ailanthone | 4.68 | 3 | Negative | 375.112 | 301.05 | 16.89 | 96 | 197.554 |
| 13,18-dehydroglaucarubinone | 7.07 | 3 | Negative | 491.242 | 473.133 | 12.81 | 185 | 96.001 |
| Glaucarubinone | 7.29 | 3 | Negative | 493.091 | 301.133 | 18.82 | 164 | 96.001 |
| Neoquassin | 9.24 | 3 | Positive | 391.095 | 206.967 | 25.55 | 174 | 96.001 |
| Quassin | 9.22 | 3 | Positive | 389.079 | 222.967 | 20.75 | 185 | 96.001 |
| Grandilactone | 9.48 | 3 | Positive | 477.179 | 359.05 | 16.17 | 151 | 96.001 |
| 2-acetyl-glaucarubinone | 9.89 | 3 | Negative | 535.101 | 301.133 | 15.81 | 180 | 96.001 |

**Table S2:** Mean concentration ± standard error of the mean (SEM) of quassinoids in different types of samples and using different rearing methods or different host plants.

| Sample Type | Quassinoid | Mean Concentration ± SEM (ppb) |
| --- | --- | --- |
| River birch | 13,18-dehydroglaucarubinone | 402.3 ± 148.9 |
|  | 2'-acetylglaucarubinone | 0 ± 0 |
|  | Ailanthone | 14.28 ± 3.22 |
|  | Glaucarubinone | 4.42 ± 1.29 |
|  | Grandilactone A | 4.06 ± 2.01 |
|  | Neoquassin | 4.85 ± 2.11 |
|  | Quassin | 4.82 ± 2.10 |
| Grape | 13,18-dehydroglaucarubinone | 389.0 ± 236.1 |
|  | 2'-acetylglaucarubinone | 0 ± 0 |
|  | Ailanthone | 1,409 ± 1,050 |
|  | Glaucarubinone | 164.1 ± 118.8 |
|  | Grandilactone A | 39.19 ± 25.79 |
|  | Neoquassin | 111.6 ± 94.08 |
|  | Quassin | 111.1 ± 93.59 |
| Silver maple | 13,18-dehydroglaucarubinone | 1.61 ± 0.368 |
|  | 2'-acetylglaucarubinone | 0.437 ± 0.437 |
|  | Ailanthone | 11.5 ± 3.08 |
|  | Glaucarubinone | 10.3 ± 7.38 |
|  | Grandilactone A | 15.5 ± 9.58 |
|  | Neoquassin | 3.53 ± 1.83 |
|  | Quassin | 3.51 ± 1.82 |
| Black walnut | 13,18-dehydroglaucarubinone | 6.45 ± 0.695 |
|  | 2'-acetylglaucarubinone | 2.36 ± 0.957 |
|  | Ailanthone | 11.6 ± 4.38 |
|  | Glaucarubinone | 6.41 ± 2.68 |
|  | Grandilactone A | 10.8 ± 3.37 |
|  | Neoquassin | 0.508 ± 0.319 |
|  | Quassin | 0.505 ± 0.318 |
| Weeping willow | 13,18-dehydroglaucarubinone | 14.0 ± 10.5 |
|  | 2'-acetylglaucarubinone | 0.379 ± 0.379 |
|  | Ailanthone | 72.9 ± 46.5 |
|  | Glaucarubinone | 154.7 ± 115.7 |
|  | Grandilactone A | 7.68 ± 5.92 |
|  | Neoquassin | 18.19 ± 9.29 |
|  | Quassin | 18.10 ± 9.24 |
| Field collected *A. altissima* phloem 2022 | 13,18-dehydroglaucarubinone | 34,983 ± 7,517 |
|  | 2'-acetylglaucarubinone | 421,542 ± 50,979 |
|  | Ailanthone | 13,557 ± 2,761 |
|  | Glaucarubinone | 138,366 ± 29,894 |
|  | Grandilactone A | 0 ± 0 |
|  | Neoquassin | 0 ± 0 |
|  | Quassin | 34,983 ± 7,517 |
| Field collected *A. altissima* phloem 2023 | 13,18-dehydroglaucarubinone | 0 ± 562.5 |
|  | 2'-acetylglaucarubinone | 968.0 ± 441.1 |
|  | Ailanthone | 21,416 ± 2,158 |
|  | Glaucarubinone | 1,511 ± 661.0 |
|  | Grandilactone A | 2,915 ± 283.6 |
|  | Neoquassin | 0 ± 0.318 |
|  | Quassin | 3,227 ± 0.986 |
| Potted *A. altissima* phloem | 13,18-dehydroglaucarubinone | 761.9 ± 199.2 |
|  | 2'-acetylglaucarubinone | 898.4 ± 76.55 |
|  | Ailanthone | 12,111 ± 2,134 |
|  | Glaucarubinone | 765.0 ± 214.5 |
|  | Grandilactone A | 1,075 ± 253.5 |
|  | Neoquassin | 0 ± 0 |
|  | Quassin | 51.73 ± 0 |
| Field collected first instar *L. delicatula* | 13,18-dehydroglaucarubinone | 55,199 ± 7,414 |
|  | 2'-acetylglaucarubinone | 8,542 ± 1,261 |
|  | Ailanthone | 294,383 ± 35,909 |
|  | Glaucarubinone | 68,119 ± 15,531 |
|  | Grandilactone A | 302.7 ± 31.34 |
|  | Neoquassin | 0 ± 0.318 |
|  | Quassin | 60.22 ± 0.984 |
| Field collected second instar *L. delicatula* | 13,18-dehydroglaucarubinone | 11,354 ± 3,273 |
|  | 2'-acetylglaucarubinone | 2,380 ± 502.1 |
|  | Ailanthone | 65,712 ± 1,189 |
|  | Glaucarubinone | 18,760 ± 5,874 |
|  | Grandilactone A | 273.1 ± 30.36 |
|  | Neoquassin | 1.08 ± 0.772 |
|  | Quassin | 10.35 ± 0 |
| Field collected third instar *L. delicatula* | 13,18-dehydroglaucarubinone | 11,030 ± 1,249 |
|  | 2'-acetylglaucarubinone | 2,492 ± 149.9 |
|  | Ailanthone | 82,697 ± 9,178 |
|  | Glaucarubinone | 19,112 ± 2,915 |
|  | Grandilactone A | 647.2 ± 115.8 |
|  | Neoquassin | 2.16 ± 1.31 |
|  | Quassin | 17.09 ± 0.163 |
| Field collected fourth instar *L. delicatula* | 13,18-dehydroglaucarubinone | 4,813 ± 1,175 |
|  | 2'-acetylglaucarubinone | 1,753 ± 240.5 |
|  | Ailanthone | 58,500 ± 2,228 |
|  | Glaucarubinone | 8,320 ± 2,085 |
|  | Grandilactone A | 614.5 ± 80.99 |
|  | Neoquassin | 1.03 ± 0.986 |
|  | Quassin | 10.35 ± 0 |
| Field collected adult male and female *L. delicatula* | 13,18-dehydroglaucarubinone | 19,090 ± 11,746 |
|  | 2'-acetylglaucarubinone | 982.0 ± 565.8 |
|  | Ailanthone | 371,881 ± 223,932 |
|  | Glaucarubinone | 52,326 ± 31,357 |
|  | Grandilactone A | 1,889 ± 569.9 |
|  | Neoquassin | 0 ± 24.13 |
|  | Quassin | 419.4 ± 74.74 |
| Field collected *L. delicatula* eggs | 13,18-dehydroglaucarubinone | 1,114 ± 447.2 |
|  | 2'-acetylglaucarubinone | 4,942 ± 1,208 |
|  | Ailanthone | 46,776 ± 9,686 |
|  | Glaucarubinone | 20,873 ± 8,288 |
|  | Grandilactone A | 2,574 ± 596.0 |
|  | Neoquassin | 0 ± 116.4 |
|  | Quassin | 1,549 ± 348.0 |
| 1^st^ instar *L. delicatula* greenhouse reared on *A. altissima* | 13,18-dehydroglaucarubinone | 24,687 ± 6,270 |
|  | 2'-acetylglaucarubinone | 17,243 ± 529.4 |
|  | Ailanthone | 100,627 ± 35,567 |
|  | Glaucarubinone | 85,717 ± 18,308 |
|  | Grandilactone A | 2,145 ± 962.3 |
|  | Neoquassin | 0 ± 0.873 |
|  | Quassin | 28.28 ± 2.70 |
| 2^nd^ instar *L. delicatula* greenhouse reared on *A. altissima* | 13,18-dehydroglaucarubinone | 80,359 ± 42,039 |
|  | 2'-acetylglaucarubinone | 57,428 ± 20,538 |
|  | Ailanthone | 194,725 ± 104,264 |
|  | Glaucarubinone | 171,619 ± 53,726 |
|  | Grandilactone A | 8,793 ± 3,327 |
|  | Neoquassin | 0.633 ± 2.71 |
|  | Quassin | 53.80 ± 20.27 |
| 3^rd^ instar *L. delicatula* greenhouse reared on *A. altissima* | 13,18-dehydroglaucarubinone | 31,476 ± 10,440 |
|  | 2'-acetylglaucarubinone | 22,539 ± 6,149 |
|  | Ailanthone | 95,648 ± 30,955 |
|  | Glaucarubinone | 50,084 ± 14,398 |
|  | Grandilactone A | 594.9 ± 232.7 |
|  | Neoquassin | 0 ± 0.749 |
|  | Quassin | 16.61 ± 1.92 |
| 4^th^ instar *L. delicatula* greenhouse reared on *A. altissima* | 13,18-dehydroglaucarubinone | 17,058 ± 7,492 |
|  | 2'-acetylglaucarubinone | 12,979 ± 4,779 |
|  | Ailanthone | 42,507 ± 16,944 |
|  | Glaucarubinone | 30,696 ± 11,438 |
|  | Grandilactone A | 280.8 ± 110.7 |
|  | Neoquassin | 0 ± 0.267 |
|  | Quassin | 15.40 ± 0.826 |
| Adult *L. delicatula* greenhouse reared on *A. altissima* | 13,18-dehydroglaucarubinone | 8,481 ± 3,850 |
|  | 2'-acetylglaucarubinone | 16,410 ± 5,742 |
|  | Ailanthone | 38,850 ± 13,927 |
|  | Glaucarubinone | 53,432 ± 21,024 |
|  | Grandilactone A | 2752 ± 1,238 |
|  | Neoquassin | 0 ± 2.29 |
|  | Quassin | 35.17607 ± 4.93 |
| *L. delicatula* eggs from adults greenhouse reared on *A. altissima* | 13,18-dehydroglaucarubinone | 16,154 ± 3,045 |
|  | 2'-acetylglaucarubinone | 23,176 ± 1,545 |
|  | Ailanthone | 55,244 ± 6,064 |
|  | Glaucarubinone | 89,474 ± 8,613 |
|  | Grandilactone A | 6,106 ± 1,060 |
|  | Neoquassin | 4.41 ± 5.37 |
|  | Quassin | 19.31 ± 0.845 |
| 1^st^ instar *L. delicatula* reared without *A. altissima* | 13,18-dehydroglaucarubinone | 11,508 ± 8,073 |
|  | 2'-acetylglaucarubinone | 1,622 ± 1,309 |
|  | Ailanthone | 45,675 ± 28,467 |
|  | Glaucarubinone | 35,661 ± 21,564 |
|  | Grandilactone A | 535.2 ± 221.4 |
|  | Neoquassin | 0 ± 125.9 |
|  | Quassin | 669.6 ± 389.8 |
| 2^nd^ instar *L. delicatula* reared without *A. altissima* | 13,18-dehydroglaucarubinone | 1,700 ± 1,449 |
|  | 2'-acetylglaucarubinone | 2,010 ± 1,305 |
|  | Ailanthone | 41,711 ± 24,475 |
|  | Glaucarubinone | 8,482 ± 4,698 |
|  | Grandilactone A | 577.6 ± 228.5 |
|  | Neoquassin | 0 ± 131.7 |
|  | Quassin | 547.3 ± 407.7 |
| 3^rd^ instar *L. delicatula* reared without *A. altissima* | 13,18-dehydroglaucarubinone | 804.8 ± 744.0 |
|  | 2'-acetylglaucarubinone | 153.5 ± 141.0 |
|  | Ailanthone | 17,291 ± 12,785 |
|  | Glaucarubinone | 5,232 ± 3,636 |
|  | Grandilactone A | 84.10 ± 28.08 |
|  | Neoquassin | 0 ± 14.14 |
|  | Quassin | 126.8 ± 43.79 |
| 4^th^ instar *L. delicatula* reared without *A. altissima* | 13,18-dehydroglaucarubinone | 463.9 ± 240.8 |
|  | 2'-acetylglaucarubinone | 210.3 ± 130.3 |
|  | Ailanthone | 1,840 ± 188.1 |
|  | Glaucarubinone | 2,660 ± 829.9 |
|  | Grandilactone A | 52.77 ± 21.96 |
|  | Neoquassin | 0 ± 1.70 |
|  | Quassin | 28.48 ± 5.26 |
| Adult *L. delicatula* reared without *A. altissima* | 13,18-dehydroglaucarubinone | 2,493 ± 1,224 |
|  | 2'-acetylglaucarubinone | 982.3 ± 217.2 |
|  | Ailanthone | 41,570 ± 16,765 |
|  | Glaucarubinone | 22,349 ± 12,468 |
|  | Grandilactone A | 833.2 ± 265.2 |
|  | Neoquassin | 0 ± 10.56 |
|  | Quassin | 86.22 ± 32.72 |
| *L. delicatula* eggs from females reared without *A. altissima* | 13,18-dehydroglaucarubinone | 475.5 ± 164.6 |
|  | 2'-acetylglaucarubinone | 853.9 ± 193.8 |
|  | Ailanthone | 2,833 ± 739.6 |
|  | Glaucarubinone | 7,023 ± 2,000 |
|  | Grandilactone A | 473.8 ± 192.1 |
|  | Neoquassin | 0 ± 4.91 |
|  | Quassin | 45.52 ± 15.21 |
| *L. delicatula* cuticle | 13,18-dehydroglaucarubinone | 7,744 ± 353.0 |
|  | 2'-acetylglaucarubinone | 12,075 ± 7,440 |
|  | Ailanthone | 56,182 ± 2,053 |
|  | Glaucarubinone | 10,335 ± 586.2 |
|  | Grandilactone A | 8,004 ± 1,114 |
|  | Neoquassin | 5.80 ± 1.01 |
|  | Quassin | 5.77 ± 1.01 |
| *L. delicatula* fat body | 13,18-dehydroglaucarubinone | 7,773 ± 651.9 |
|  | 2'-acetylglaucarubinone | 1,864 ± 236.1 |
|  | Ailanthone | 43,327 ± 3,711 |
|  | Glaucarubinone | 8,925 ± 855.2 |
|  | Grandilactone A | 2,460 ± 426.3 |
|  | Neoquassin | 1.95 ± 0.265 |
|  | Quassin | 1.94 ± 0.263 |
| *L. delicatula* gut | 13,18-dehydroglaucarubinone | 194,251 ± 22,555 |
|  | 2'-acetylglaucarubinone | 351,746 ± 327,834 |
|  | Ailanthone | 1,457,108 ± 118,386 |
|  | Glaucarubinone | 222,533 ± 29,904 |
|  | Grandilactone A | 26,873 ± 5,746 |
|  | Neoquassin | 0 ± 0 |
|  | Quassin | 0 ± 0 |
| *L. delicatula* ovary | 13,18-dehydroglaucarubinone | 17,265 ± 3,241 |
|  | 2'-acetylglaucarubinone | 10,740 ± 6,516 |
|  | Ailanthone | 76,981 ± 15,216 |
|  | Glaucarubinone | 18,230 ± 3,356 |
|  | Grandilactone A | 3,311 ± 997.4 |
|  | Neoquassin | 6.24 ± 2.29 |
|  | Quassin | 6.21 ± 2.28 |
| *L. delicatula* salivary gland | 13,18-dehydroglaucarubinone | 295,231 ± 45,615 |
|  | 2'-acetylglaucarubinone | 2,141,152 ± 2,098,060 |
|  | Ailanthone | 3,415,981 ± 292,322 |
|  | Glaucarubinone | 416,208 ± 70,583 |
|  | Grandilactone A | 73,592 ± 12,529 |
|  | Neoquassin | 88.69 ± 37.07 |
|  | Quassin | 88.22 ± 36.87 |
| *L. delicatula* other tissues | 13,18-dehydroglaucarubinone | 14,999 ± 2,147 |
|  | 2'-acetylglaucarubinone | 9,175 ± 4,805 |
|  | Ailanthone | 87,193 ± 11,495 |
|  | Glaucarubinone | 18,416 ± 2688 |
|  | Grandilactone A | 5,556 ± 1,329 |
|  | Neoquassin | 6.43 ± 1.24 |
|  | Quassin | 6.40 ± 1.23 |

*All *L. delicatula* without access to *A. altissima* were reared in enclosures
